# Enhancing long-term smoking abstinence among individuals with a history of cervical intraepithelial neoplasia or cervical cancer (Project ACCESS): protocol for a randomized clinical trial

**DOI:** 10.1186/s12889-023-16189-3

**Published:** 2023-07-04

**Authors:** Jennifer I. Vidrine, Bethany Shorey Fennell, Vani N. Simmons, Steven K. Sutton, Sarah R. Jones, Honor W. Woodward, Charles E. Hoogland, Damon J. Vidrine

**Affiliations:** 1 Department of Health Outcomes and Behavior, Moffitt Cancer Center, 12902 Magnolia Dr, Tampa, FL 33612 USA; 2 Department of Oncologic Sciences, Morsani College of Medicine, University of South Florida, Tampa, FL USA; 3 Department of Psychology, University of South Florida, Tampa, FL USA; 4 Department of Biostatistics and Bioinformatics, Moffitt Cancer Center, Tampa, FL USA

**Keywords:** Cervical cancer, Cervical intraepithelial neoplasia, Cervical dysplasia, Cancer, Smoking, Smoking cessation, Tobacco cessation

## Abstract

**Background:**

The prevalence of smoking among cervical cancer survivors is high and evidence-based smoking cessation interventions are critically needed. This paper describes the study design, methods, and data analysis plans for a randomized clinical trial (RCT) designed to evaluate the efficacy of a novel, personally tailored SMS-delivered text-based digital treatment adjuvant designed to enhance the long-term efficacy of a “**M**otivation **A**nd **P**roblem-**S**olving” (**MAPS**) approach for smoking cessation among individuals with a history of cervical intraepithelial neoplasia (CIN) or cervical cancer. MAPS is a phone counseling approach designed to facilitate long-term abstinence that comprises 6 counseling calls over 12 months. The current trial is evaluating the efficacy of **MAPS+**, which comprises all MAPS components plus a 24-month digital treatment adjuvant. This trial represents a logical extension of our previous RCT, which compared the efficacy of MAPS to a quitline control condition and found that MAPS resulted in greater than a 2-fold increase in smoking abstinence at 12 months (i.e., 26.4% vs. 11.9%). This treatment effect was no longer significant at 18 months, suggesting that efficacy dissipated as time from the end of treatment increased. The primary aim of the current trial is to compare the efficacy of MAPS + and ST in facilitating long-term abstinence.

**Methods:**

Individuals who smoke and have a history of cervical cancer or CIN (N = 340) are recruited throughout Florida and randomly assigned to **Standard Treatment** [**ST**] or **MAPS+**. ST participants are electronically connected with the Florida Quitline. MAPS + consists of 6 proactive MAPS-based counseling calls over 12 months plus the novel, personally tailored, text message-based treatment adjuvant delivered over 24 months. All participants receive 12 weeks of combination nicotine replacement therapy (patch and lozenge) and are followed for 24 months. Participant recruitment commenced in December 2022 and is ongoing.

**Discussion:**

This study builds on promising results from our recent trial which found that MAPS was associated with substantially higher abstinence from smoking at the end of the 12-month treatment period. Finding that this low-burden, personally tailored digital treatment adjuvant improves the long-term efficacy of MAPS would have important clinical and public health implications.

**Trial registration:**

Clinical Trials Registry NCT05645146; https://clinicaltrials.gov/ct2/show/NCT05645146; Registered on December 9, 2022.

## Background

In the presence of HPV, smoking is a primary risk factor for cervical intraepithelial neoplasia (CIN) – the immediate precursor to cervical cancer – and cervical cancer [1–5]. Continuing to smoke after a cancer diagnosis is associated with poor treatment response, increased risk of recurrence, second primary cancers and other diseases [3, 6–10]. In the US, there are estimated to be nearly 300,000 cervical cancer survivors [11]. Furthermore, approximately 200,000 individuals are diagnosed with CIN each year, [12] which places them at substantially elevated risk of developing cervical cancer. An alarming one-third of cervical cancer survivors report current smoking, a prevalence that is higher than among any other subgroup of cancer survivors [13]. Smoking cessation among individuals with a history of CIN or cervical cancer is a critically important clinical and public health issue. With few exceptions, [14, 15] smoking cessation interventions for the general population of cancer survivors have failed to demonstrate efficacy [13, 16]. Thus, there is a pressing need for efficacious, cost-effective, and sustainable interventions with broad dissemination potential for survivors of CIN and cervical cancer. The current study was designed to help fill this need.

Other than our previous trial, [15] no tobacco cessation studies that we know of have specifically targeted individuals with a history of CIN or cervical cancer, even though these populations have unique characteristics which warrant a targeted approach. Multiple studies have documented that cervical cancer survivors suffer moderate to poor health-related quality of life [17] and longstanding psychosocial sequelae including anxiety, depression, stress, relationship issues, and difficulty with sexual functioning [17–21]. Furthermore, cervical cancer survivors with lower SES and limited social support are at even greater risk for poor outcomes [17, 22, 23]. Because our Motivation And Problem Solving+ (MAPS+) approach is built around a Wellness Program that addresses life events, stressors, and other concerns (e.g., anxiety, stress, fear of cancer or cancer recurrence, family conflicts and relationship issues), it is especially well-suited to addressing concerns of particular relevance to survivors of CIN or cervical cancer.

## MAPS + overview and rationale

MAPS is a holistic, dynamic framework for behavior change that integrates treatment elements from both motivational interviewing [24, 25] and social cognitive theory [26–28]. It is designed for all individuals regardless of their readiness to quit, and specifically targets motivation, agency/self-efficacy, and stress/negative affect. Counselors are trained to carefully assess and respond to changes in motivation so that treatment strategies are appropriately matched in the moment. MAPS is built around a Wellness Program that, in addition to focusing on smoking, addresses life events, stressors, and other immediate concerns. By addressing the larger context in which smoking cessation occurs, not only are many of the barriers to successfully quitting smoking addressed, but treatment engagement is also likely to be enhanced because individuals perceive that the counselors care about them as whole people and are not solely interested in their smoking behavior.

Our team recently completed a trial that evaluated the efficacy of MAPS among individuals with a history of CIN or cervical cancer and results indicated that MAPS was associated with a greater than two-fold increase in smoking abstinence at 12 months compared to a quitline treatment control condition. Unfortunately, the large treatment effect observed at 12 months diminished over time and was no longer significant at 18 months [15]. The personally tailored text-based digital treatment adjuvant being evaluated in the current study is intended to enhance long-term abstinence through extending support and reinforcing motivational enhancement and coping skills training provided by the MAPS counselors. Text message content was designed to emphasize and reinforce specific MAPS-based treatment content areas such as managing cravings, practicing coping skills, handling ambivalence about quitting smoking and maintaining abstinence, stress management, relationship issues, and survivorship-specific concerns. Furthermore, this treatment content is tailored to participants’ need for support in specific areas, their smoking status, and their self-reported levels of motivation, agency and stress/negative affect. The purpose of the 24-month digital treatment adjuvant is to extend and enhance participants’ relationships with their MAPS counselors. This is achieved by training counselors to encourage participants to utilize the support and information provided by the text messages between counseling calls and after counseling has ended. Counselors are also trained to refer participants back to specific text content for acute challenges such as managing cravings, anticipating and planning for “high risk” situations and refreshing coping skills.

This paper describes the research design, methods, and data analysis plans for an ongoing randomized clinical trial (RCT) designed to evaluate the efficacy of MAPS + in facilitating smoking cessation among survivors of CIN or cervical cancer. The primary aim of the trial is to compare the efficacy of MAPS + in facilitating smoking cessation with standard treatment (ST). A secondary aim is to evaluate motivation for smoking cessation, agency, and stress/negative affect as prospective mediators of the difference in abstinence between the MAPS + and ST treatment groups. Whereas each is a likely mechanism for both interventions, the relative importance may differ. For example, higher levels of quit motivation may mediate abstinence in ST, whereas higher levels of agency may mediate abstinence in MAPS+.

## Methods and design

### Study design overview

**Fig. 1:**
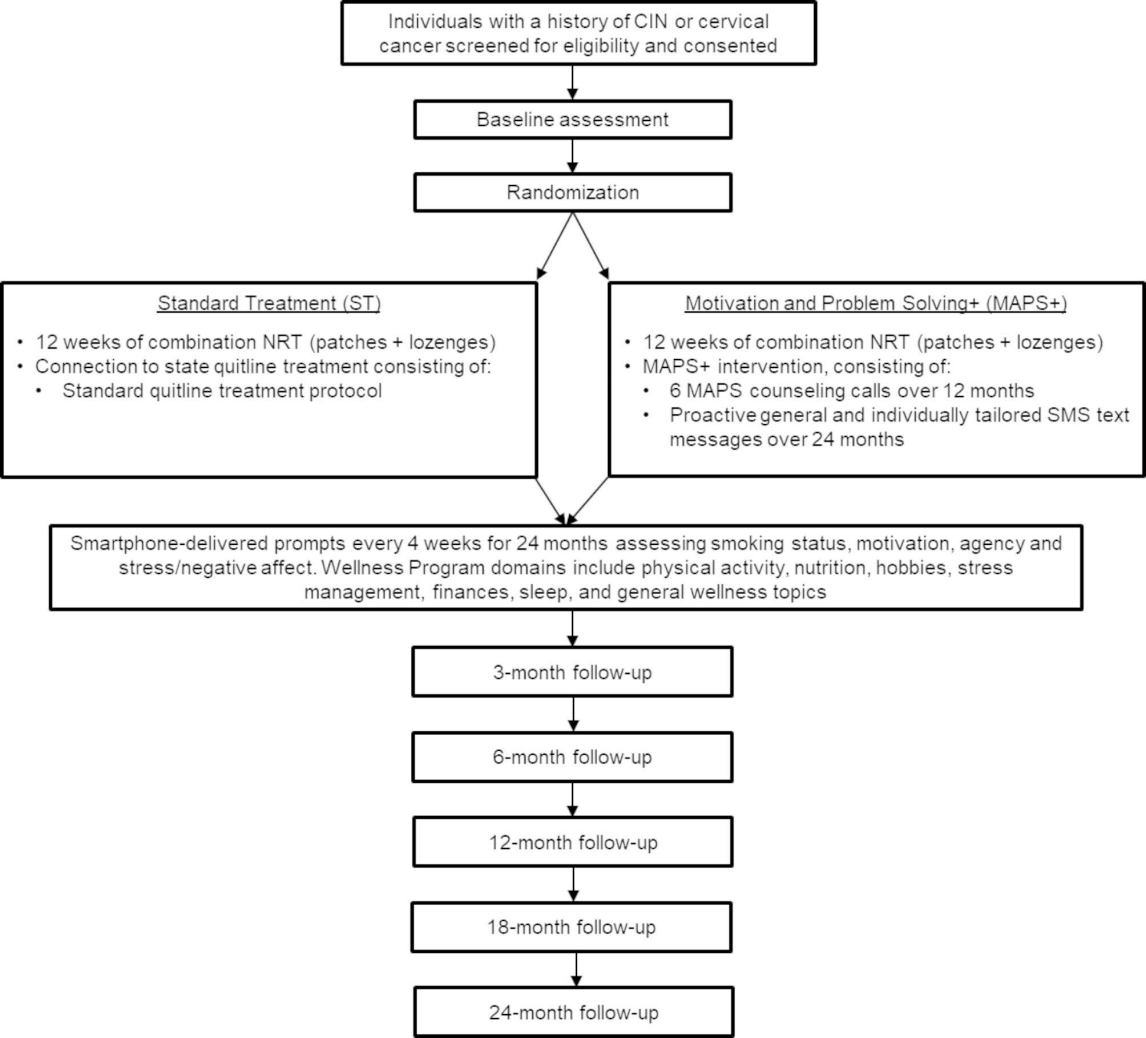
Study flow

A 2-group RCT is underway to compare the efficacy of MAPS + vs. ST (Fig. 1).

ST participants are electronically connected with Tobacco Free Florida (i.e., the Florida Quitline). MAPS + consists of 6 proactive MAPS-based phone counseling sessions over 12 months plus a novel digital treatment adjuvant, which is delivered over 24 months. All participants receive 12 weeks of combination nicotine replacement therapy (patches + lozenges). Assessments are completed at baseline, 3, 6, 12, 18 and 24 months following baseline. All assessments are completed remotely (over the phone or via a secure REDCap link). In addition, brief REDCap-based smartphone assessments are delivered every 30 days, which are used to drive the personalized text-based treatment content in the MAPS + condition, are administered throughout the 24-month treatment period. These assessments are also delivered to ST participants to ensure that both treatment conditions are matched on assessment burden. The primary outcome is self-reported 7-day point prevalence abstinence over time throughout the 24-month assessment period [29, 30]. Secondary outcomes include additional smoking-related outcomes (i.e., biochemically verified abstinence, length of abstinence, continuous abstinence, cigarettes per day, quit attempts).

It is hypothesized that self-reported, 7-day point prevalence abstinence rates will be greater in MAPS + than in ST over time throughout the 24-month assessment period. For secondary outcomes, it is hypothesized that MAPS+ (vs. ST) participants will have higher biochemically confirmed abstinence rates at 24 months and across all assessments, make more quit attempts, report longer periods of abstinence, and report smoking fewer cigarettes per day across all assessments throughout the 24-month study period.

### Recruitment and participants

Participant recruitment began in December 2022. Participants (target sample size = 340) are recruited through internet advertisements (i.e., Facebook, Instagram, Google pay-per-click ads, Craigslist) and internet-based, healthcare provider or community referrals (e.g., ClinicalTrials.gov, flyers, word-of-mouth). Inclusion criteria are: (1) ≥ 18 years of age; (2) ≥ 100 lifetime cigarettes; (3) English-speaking; (4) self-report of smoking ≥ 1 cigarette in past 30 days; (5) history of cervical cancer or CIN; (6) working smartphone; (7) valid home address; and (8) reside in Florida at the time of study enrollment. Exclusion criteria are: (1) medical condition precluding NRT; (2) currently receiving behavioral or pharmacological tobacco treatment; (3) household member enrolled in the study.

### Procedures

This study was reviewed and approved by Advarra, the H. Lee Moffitt Cancer Center institutional review board (IRB) and is registered on ClinicalTrials.gov (NCT05645146). After completion of a brief online pre-screener, potential participants are contacted by research staff via phone to complete a more detailed screening. All eligible individuals are invited to participate. A detailed description of the study is provided, and verbal informed consent is obtained. Individuals who decline or are ineligible are referred to other cessation programs. Individuals who meet all eligibility criteria and consent to participating in the study complete the baseline assessment either over the phone with a research coordinator or via a secure electronic REDCap link sent via text message or email based on their preference. Following completion of the baseline assessment, participants are randomized to MAPS + or ST using a 1:1 ratio with a block size of 4 following stratification on the following variables: daily vs. nondaily smoking; CIN and early cancer stages (Stages I and Stage II) vs. advanced cancer stages (Stage III and Stage IV), and pending or in active treatment vs. completed treatment. The randomization sequences were created by the study statistician and implemented in REDCap.

Assessments occur at baseline, 3, 6, 12, 18 and 24 months and take approximately 20 min to complete (see Table 1). Participants are given the option to complete the assessments via phone or via a hyperlink as a REDCap-administered self-assessment. Compensation is provided after each assessment is completed (6 assessments x $30 = $180). Participants who report abstinence at any follow-up point are mailed a cotinine test. Compensation for returning cotinine tests is $30 per assessment.

### Intervention conditions

#### Standard treatment

Participants randomized to ST are electronically connected with Tobacco Free Florida by the study team. Treatment consists of the standard treatment offered by Tobacco Free Florida, which is consistent with the Treating Tobacco Use and Dependence Clinical Practice Guideline [31, 32] in that treatment content is drawn from cognitive-behavioral and motivational interviewing techniques.

#### Motivation and problem solving+ (MAPS+)

MAPS + includes (1) 6 proactive, flexibly-offered telephone counseling sessions delivered over 12 months, and (2) proactive, text-based general and personally tailored treatment content delivered via SMS throughout the entire 24-month treatment period. The timing of the telephone counseling sessions during the first 12 months of the study period is determined jointly by the participant and their MAPS counselor. For example, participants struggling with maintaining abstinence may request several calls in a shorter period of time to get through the problematic period, whereas others prefer a less compressed counseling schedule and may need less frequent help. Each call lasts 30 min on average.

MAPS utilizes an innovative combination of motivational enhancement and social cognitive techniques, is designed for all individuals regardless of their readiness to quit, and specifically targets motivation, agency, and negative affect/stress. Counselors are trained to carefully assess and respond to changes in motivation so that treatment strategies are appropriately matched in the moment.

*General, non-tailored MAPS + text message content*. The general (i.e., non-tailored) text-based treatment content delivered via SMS is designed to provide information regarding general smoking cessation strategies and support as well as messages specifically related to participants’ diagnoses (i.e., CIN or cervical cancer). Treatment content delivery is less intensive in the first 12 months of the study period and increases slightly during months 13 through 24 to compensate for the end of human-delivered MAPS counseling support. Specifically, during the first 12 months, which runs concurrently with the human-delivered MAPS counseling delivery, participants receive 2 non-tailored text messages per week. In months 13 through 24, the frequency is increased to 3 non-tailored messages each week.

*Personally tailored MAPS + text message content*. In addition to the general treatment content delivered via SMS, MAPS + participants receive personalized text message-based treatment content which is delivered for the first week of every 30-day period throughout the entire 24-month treatment period. This personalized treatment content is tailored on participants’ self-reported smoking-status and their self-reported levels of motivation, agency, and negative affect/stress. Each construct is assessed using a single item. Specifically, every 30 days, participants receive smartphone-delivered prompts generated by REDCap to assess their need for support with both smoking-relevant issues and broader life issues including Wellness Program topics. In addition to asking about smoking status, motivation, agency and negative affect/stress, each monthly series of questions asks participants about key domains related to their Wellness Programs in which they may need support including physical activity, nutrition, hobbies, stress management, finances, sleep, and general wellness topics. As described above, the text-based treatment content is personalized on these 4 dimensions (i.e., smoking status, motivation, agency, and negative affect/stress. Participants can also choose to receive Wellness Program content in up to three topic areas. Participants who do not select specific Wellness Program topics receive text content related to general wellness topics. Throughout the entire 24-month study period, participants receive between 5 and 7 personally tailored text messages the first week of every 30-day period. For the remaining weeks, participants receive up to 3 personally tailored wellness program messages.

#### MAPS counselor qualifications and training

We have adopted the therapist selection criteria employed in major clinical trials of MI-based approaches: (1) master’s degree in counseling, psychology, social work, or a related field, and (2) 2 + years of clinical experience [33]. This, along with our training and ongoing monitoring, helps to ensure that the delivered treatment is of the highest quality. Counselors receive 20 h of MAPS training initially. Training continues until the counselor reaches performance criteria for competence and adherence to the protocol. Counselors participate in 1–2 h “booster” training sessions every 2 months. In addition, the counselors receive regular clinical supervision to discuss participants’ progress and to support adherence to the treatment manual.

#### MAPS counseling fidelity and monitoring

To monitor deviation or drift from the protocol, counseling calls are digitally recorded and encrypted. A random sample of 10% of calls is coded to ensure adequate adherence. A counselor who falls below performance criteria will receive additional training. The Motivational Interviewing Treatment Integrity Manual (MITI, 4th edition) [34] has empirically validated reliability and validity and is used to code sessions and ensure treatment fidelity. The MITI works well for ensuring that counselors are following the protocol and utilizing the general motivational interviewing spirit. In addition, the MITI is modified slightly to include coding of discussions around social cognitive/problem solving strategies and transitions between motivational enhancement and problem-solving. Weekly reports are reviewed to track call completion rates.

### Nicotine replacement therapy

All study participants receive 12 weeks of combination nicotine replacement therapy (patches + lozenges). Nicotine patches provide a low, constant level of nicotine, which attenuates nicotine withdrawal symptoms, and lozenges help with managing acute cravings to smoke. When combined with behavioral treatment, NRT doubles the odds of successfully quitting [26]. All participants receive educational materials describing potential side effects, proper use of the patch, and an illustration demonstrating the proper placement of the patch on the body. The nicotine patch and lozenge regimens are based on each participant’s self-reported smoking rate. Participants who smoke > 10 cigarettes/day receive 8 weeks of 21 mg patches, 2 weeks of 14 mg patches, 2 weeks of 7 mg patches, and 12 weeks of 2 mg lozenges. Those who smoke < = 10 cigarettes/day receive 8 weeks of 14 mg patches, 4 weeks of 7 mg patches, and 12 weeks of 2 mg lozenges.

### Measures

All study measures were selected based on established reliability and validity. If measures with established psychometric properties were not available, those chosen were required to have at least face validity. Assessments are given at baseline, 3, 6, 12, 18 and 24 months and require approximately 20 min to complete (see Table 1 for a list of all measures and assessment schedule).

**Table 1:** Study measures and assessment schedule

Measure	Baseline Assessments	Smartphone Assessments (every 30 days)	Follow-Up Assessments (3, 6, 12, 18 and 24 months)
**DEMOGRAPHICS, SMOKING, SUBSTANCE USE, HEALTH HISTORY**			
Demographics and Smoking History	X		
History of CIN or Cervical Cancer	X		
Medical Comorbidities	X		
Cancer Patient – Tobacco Use Questionnaire (C-TUQ)	X		X
Heaviness of Smoking Index (HSI)	X		
Wisconsin Index of Smoking Dependence (WISDM-37)			X
Substance Use History	X		
Alcohol Use History (NIAAA)	X		X
Subjective Social Status	X		X
Health Literacy	X		
**MOTIVATION**			
Contemplation Ladder	X		X
Reasons for Quitting (Intrinsic and Extrinsic Motivation)	X		X
**AGENCY: SENSE OF CONTROL, SELF-EFFICACY, COPING BEHAVIOR**			
Sense of Control	X	X	X
Self-Efficacy Scale	X	X	X
Coping Inventory	X		X
**STRESS, NEGATIVE AFFECT, PSYCHOLOGICAL DISTRESS**			
Perceived Stress Scale (PSS-4)	X	X	X
Positive and Negative Affect Scale (PANAS)	X		X
Patient Health Questionnaire (PHQ-8)	X		X
**SMOKING ABSTINENCE**			
Smoking Status (SRNT)	X	X	X
**PATIENT REPORTED OUTCOMES**			
Functional Assessment of Cancer Therapy – Cervix Cancer (FACT-Cx)	X		X
Fear of Cancer Recurrence Inventory-Short Form*	X		X
**SATISFACTION WITH TREATMENT**			
Client Satisfaction Questionnaire (CSQ)			X**

*Modified for CIN participants as ‘Fear of Cancer’

**Only administered at 12- and 24-month follow-ups

Participants who report being abstinent from smoking at any follow-up assessment are mailed a prepaid envelope, instructions for providing a saliva sample, and a saliva collection kit. Research staff guide participants in providing the saliva samples and in returning the samples via mail.

#### Demographics, smoking history, alcohol and substance use, and health history

Demographic variables assessed include age, race, ethnicity, education, income, gender identity, sex assigned at birth, marital status, sexual orientation, occupational status and insurance status. Smoking history is assessed by asking about years smoking, preferred brand of cigarettes, quitting history, partner smoking, and the presence of other people who smoke in the household. The use of e-cigarettes (vaping) is also assessed. History of alcohol use is assessed using NIAAA core items [35, 36]. Substance use history is assessed with three items asking about illegal substance use in the past year.

History of CIN or cervical cancer is assessed by asking about diagnostic history, dysplasia grade or cervical cancer stage at the time of diagnosis, current CIN or cervical cancer stage, time since diagnosis, treatment status and time since completion of treatment.

The *Cancer Patient Tobacco Use Questionnaire (C-TUQ)* is a 22-item self-report survey designed to capture information about tobacco use by cancer patients and cancer survivors. The items are specifically tailored to the trajectory of cancer diagnosis, treatment, and survivorship. The C-TUQ survey tool is divided into five domains and includes a Core (short form of 4 items) and an Extension. The questionnaire is intended to be administered at key time points during periods of cancer treatment and recovery [37].

Nicotine dependence is assessed using the *Heaviness of Smoking Index (HSI)*. The HSI comprises two items from the Fagerstrom Test for Nicotine Dependence (FTND): self-reported average number of cigarettes smoked per day (CPD) and time to first cigarette upon waking (TTFC). The HSI is a good indicator of nicotine dependence, has fair internal consistency and is predictive of smoking relapse [38].

*The Wisconsin Index of Smoking Dependence Motives – Short Form (WISDM-37)* comprises 37 items designed to assess 11 different motivational domains: affective enhancement, affiliative attachment, automaticity, loss of control, cognitive enhancement, craving, cue exposure/associative processes, social/environmental goads, taste/sensory properties, tolerance, and weight control [39, 40].

The *Subjective Social Status* measure consists of a 10-rung ladder designed to represent different levels that a person may occupy within society. This scale has been shown to be a reliable measure of subjective social status among diverse populations and has shown a stronger relationship with health than many objective SES measures among those populations [41–43].

*Health literacy* is assessed using a single item, “How confident are you filling out medical forms by yourself?” [44] Responses are on a 5-point scale and include the following: 1 = “not at all,” 2 = “a little bit,” 3 = “somewhat,” 4 = “quite a bit,” and 5 = “extremely.” This single health literacy item has demonstrated adequate face, construct, content, and criterion validity for identifying individuals with limited health literacy in racially/ethnically diverse populations, [44–50] and is highly correlated with more comprehensive measures of health literacy including the REALM [51] and the STOFHLA [44, 45, 47–49, 52, 53]. Regarding scoring, the “somewhat” response has been identified as the optimal cut point to classify individuals as having limited health literacy [49]. Based on this recommendation and consistent with prior research, health literacy will be dichotomized as higher vs. lower (“not at all,” “a little bit,” or “somewhat” = low; “quite a bit” or “extremely” = high) [45, 49].

#### Motivation for smoking cessation

The *Contemplation Ladder* assesses readiness to quit smoking on an 11-point “ladder” scale with steps ranging from 0, “no thought of quitting,“ to 10, “taking action to quit” (e.g., cutting down, enrolling in a program) [54].

The *Reasons for Quitting (Intrinsic and Extrinsic Motivation)* measure is a 20-item scale that assesses intrinsic (health concerns, self-control) and extrinsic (immediate reinforcement, social influence) motives for quitting smoking. Both intrinsic motives and the ratio of intrinsic to extrinsic motives have been demonstrated to predict successful smoking cessation [55].

#### Agency

*Sense of control* is measured using an 8-item index on a Likert scale ranging from 1 “strongly disagree” to 4 “strongly agree.” This scale offers an unbiased estimate of personal control/powerlessness and demonstrates high reliability (alpha = 0.83) [56].

The *Self-Efficacy Scale* is a 9-item scale reflecting the confidence of the individual that they can cope with high-risk situations without relapsing [57]. Self-efficacy is among the strongest and most-studied predictors of smoking cessation treatment outcomes [58–60].

Coping is assessed using the eight-item *Daily Coping Inventory* [61, 62]. The measure includes eight coping dimensions including situation redefinition, direct action, catharsis, acceptance, seeking social support, distraction, religion, and relaxation. The original instructions were modified to assess coping related to problems and events that occurred in the past week (rather than day), and read as follows, “Please think about the events and problems that bothered you most over the last week and decide which choices best describe you. Then choose your answer.” Each item is rated on a 5-point Likert response scale ranging from “strongly disagree” to “strongly agree.” Each coping dimension is represented by a single item. Higher scores reflect greater reliance on a particular coping dimension. Catharsis and seeking social support are associated with relatively high levels of negative affect, and acceptance is associated with relatively low levels of negative affect and relatively high levels of positive affect. Use of distraction and relaxation is associated with relatively high levels of positive affect [61, 62].

#### Stress/negative affect and psychological distress

The *Perceived Stress Scale-4 (PSS-4)* [63] is a four-item measure designed to assess the degree to which respondents find their lives to be stressful. Internal consistency is good and PSS-4 scores are predictive of relapse [64–66].

The *Positive and Negative Affect Schedule (PANAS)* comprises two mood scales: Positive Affect (PA) and Negative Affect (NA). Internal consistency for both scales is good, and negative affect scores have been among the best predictors of relapse in previous studies [58, 67, 68]. Elevated scores on these scales are indicative of greater positive affect or negative affect, respectively [69].

The *Patient Health Questionnaire* (PHQ-8) is an eight-item sale which can be used to establish provisional diagnoses for selected DSM-IV disorders. All forms are well validated and shortened forms of the PHQ, like the PHQ-*, are often used when depression and/or anxiety are of interest in a research setting. We are using the PHQ-8 in lieu of the PHQ-9 because the nature of the trial will prevent us from following up with the necessary clinical review and immediate response required when someone responds as suicidal on the final question of the PHQ-9. The PHQ-8 is scored the same as the PHQ-9 and just as sensitively detects levels of depression and anxiety [70–72].

#### Patient reported outcomes

The *Functional Assessment of Cancer Therapy – Cervix Cancer (FACT-Cx)* comprises all items from the FACT-G and the Cx subscale. The FACT-G (version 4) is a 27-item self-report quality of life measure developed and validated among cancer patients and survivors. It consists of four subscales measuring physical well-being, functional well-being, social/family well-being, and emotional well-being. Each subscale produces a score that can be aggregated into one total score. The Cx subscale contains 15 items concerning symptoms and concerns specifically related to cervical cancer developed by cervical cancer patients and clinicians. The Fact-Cx can be self-administered or used in an interview format. Individuals are asked to report how they feel today and how they have felt during the previous 7 days, with higher scores indicating better quality of life. Cronbach’s alphas for each subscale are good and have been reported as follows: psychical well-being (0.82), functional well-being (0.80), social/family well-being (0.69), emotional well-being (0.74), and total FACT-G (0.89) [73, 74].

*The Fear of Cancer Recurrence Inventory – Short Form (FCRI-SF) is a* multidimensional self-report scale designed to assess a patient’s fear of cancer recurrence [75, 76]. This measure was modified to assess fear of developing cancer for participants with a history of CIN [15, 77].

#### Smoking abstinence

*Self-report smoking abstinence* assessments are based on recommendations from the Society for Research on Nicotine and Tobacco (SRNT) [30] and include abstinence assessments at all follow-up assessments. We are evaluating two point-prevalence abstinence measures: (1) no smoking during the previous 7 days, and (2) no smoking during the previous 30 days. Point-prevalence abstinence rates (intent-to-treat) will be estimated using generalized estimating equations.

*Biochemically verified abstinence.* Biochemically verified abstinence is assessed using saliva cotinine [29]. Participants who report 7-day point-prevalence abstinence at any follow-up assessment are mailed a prepaid envelope, instructions for providing a saliva sample, and a saliva collection kit. Research staff are available to speak with participants by phone and/or email should the participant have questions about the collection process. The participant is asked to return the sample via mail using a pre-paid envelope and, upon receipt of returned saliva samples, participants are compensated $30.

*Smartphone assessments*. All participants are asked to complete brief monthly assessments through REDCap (sent via SMS) for 24 months. For participants randomized to MAPS+, these 5-item monthly assessments are used to check in and offer personally tailored treatment content delivered via text messages and matched on both (1) areas in which participants indicate a need for support, and (2) smoking status, perceived stress, motivation, and self-efficacy. Participants randomized to ST also receive monthly assessments of smoking status, perceived stress, motivation, and self-efficacy (four items) to ensure that both treatment conditions are matched on assessment contacts. However, ST participants do not receive additional treatment after completing these monthly assessments.

*Primary and secondary outcomes*. Our primary outcome is self-reported 7-day point prevalence abstinence at 24 months where participants who report no smoking in the previous 7 days are considered abstinent. This follows recommendations for community-based smoking cessation trials that do not involve in-person assessments [29, 30]. Secondary outcomes are biochemically verified abstinence, length of abstinence, continuous abstinence, cigarettes per day, and quit attempts.

### General analytic approach

We hypothesize that self-reported 7-day point prevalence abstinence rates will be greater in MAPS + than in ST. All data analyses will be performed using SAS version 9.4 with significance set at 0.05 for statistical tests of primary hypotheses. Descriptive statistics will be used for initial review of distributions and determination of needs for transformation prior to primary analyses. Group differences in baseline measures will be assessed and any variable that exhibits a group difference at p < .10 will be added as a covariate in the primary analyses.

### Statistical analysis

#### Analytic plan for primary outcome

Logistic regression will assess treatment group differences in self-reported 7-day point prevalence abstinence at 24 months. Generalized estimating equations (GEE) will fit population-averaged models of longitudinally measured self-reported 7-day point prevalence abstinence, with the main covariates of treatment group, time (months from baseline), and the interaction of group and time. The GEE analysis permits evaluation of aggregated intervention effects across assessments, changes in abstinence over time, and group differences in changes (interaction term). These models will include any covariates found to differ by group (p < .10) despite randomization. Comparable models will be applied to evaluate treatment differences for secondary outcomes.

#### Analytic plan for secondary outcomes

We propose common mechanisms (i.e., motivation, agency, and stress/negative affect) on smoking abstinence for both the MAPS + and ST treatments, but potential for differences in the relative strength of each mechanism by group. These prospective differences will be assessed via mediation analyses with intervention as the independent variable, abstinence as the outcome variable, and the hypothesized mechanisms (motivation, agency, stress/negative affect) as mediators. Whereas 24 months is the final assessment point, abstinence at earlier assessments will also be evaluated in order to capture points in time when transitions from smoking to abstinence are more likely. To assess mediation effects, we will fit both single and multiple mediator models, using the approaches of MacKinnon [78] and Preacher and Hayes,[79–81] as appropriate.

#### MAPS + evaluation

Engagement measures (e.g., number of counseling calls completed, amount of text-based treatment content received) initially will be evaluated via descriptive statistics. These variables also will be explored as predictors of smoking abstinence. First, univariate logistic regression analyses will be used to identify prospective predictors. Univariate significant predictors will be submitted to a multivariable model with backward stepwise procedures to identify the set of predictors that make a unique contribution to abstinence in the MAPS + condition.

### Missing data management

To manage missing data, multiple imputation under the Missing at Random assumption will be applied using a Markov Chain Monte Carlo method [82] via PROC MI in SAS version 9.4 given the expected large numbers of non-monotonic missing data patterns and auxiliary variables (e.g., baseline measures that predict smoking status) identified via preliminary analyses. Twenty data sets will be created. For smoking status, a post hoc adjustment [83] will be applied to implement an influence of Missing Not at Random (MNAR) (i.e., missing is due to smoking). In recent publications, [84] we have applied a small-medium effect size (Cohen’s *d* = 0.35). This approach provides better parameter estimates and tests of hypotheses than does imputing missing equals smoking. Sensitivity analyses will be performed by systematically increasing the MNAR influence in *d* = 0.15 increments to the point of missing equals smoking.

### Power and sample size

The primary statistical analyses will evaluate intervention effects on 7-day point prevalence abstinence. This will be done in two ways: (1) using logistic regression at 24 months, and (2) using GEE to assess intervention effects aggregating across all assessments. Based on results from our recently completed trial [15], abstinence rates in both groups are expected to increase over time along with an increasing difference between groups. Estimated abstinence at 24 months is 16% for ST and 29% in MAPS+. PASS 2020 [85] was used to estimate sample size/power for logistic regression analyses. With a sample size of 170 per group, power is ≥ 0.80 for logistic regression analyses at 24 months with alpha = 0.05 and a two-sided test. Using GEESIZE 3.1, [86] power ≥ 0.95 for GEE analysis of abstinence across all follow-ups. No adjustments to enrollment size will be needed to account for attrition as we will utilize multiple imputation to manage missing data.

### Data and safety monitoring plan

In line with other smoking cessation research conducted in this lab, [87] the PI will be responsible for all data monitoring and for compliance with all federal and institutional IRB policies and procedures for monitoring progress, safety, reporting of unanticipated problems or adverse events, and assuring actions resulting in suspension of the study are reported. All modifications to the protocol will be submitted for IRB approval. Summaries of all relevant discussions will be promptly disseminated to study personnel via e-mail, and retraining procedures will be implemented as needed. If necessary, appropriate modifications will be made in consultation with the designated program person at the James and Esther King Florida Biomedical Research Program, National Institutes of Health, or both.

All data collected will be kept confidential. Confidentiality will be protected by identifying all participants by ID numbers only, with all data stored and managed at Moffitt Cancer Center in a secure, HIPAA-compliant electronic database (REDCap). In addition, data storage, or data transfer if there is a request, will follow all Moffitt Cancer Center requirements for data security. When data sharing is requested, de-identified data files will be transferred on a password-protected and encrypted drive and will be maintained on institutional servers with appropriate antivirus software. Final de-identified data files will be maintained by the PI at Moffitt.

### Dissemination plan

Study findings will be disseminated to the scientific community through presentations at local, national, and international meetings and via peer-reviewed publications.

## Discussion

Given that cervical cancer survivors are more likely than other subgroups of cancer survivors to smoke, and that individuals with a history of CIN who smoke are at substantially elevated risk for developing cervical cancer, there is a pressing need to target these subpopulations with efficacious smoking cessation treatment. MAPS + builds on prior evidence that MAPS-based tobacco treatment is efficacious for individuals with a history of CIN or cervical cancer and meets the need for extended support for these vulnerable populations.

This study is the first to specifically target and address the smoking cessation treatment needs of individuals with a history of CIN or cervical cancer over an extended, two-year period. MAPS + is highly flexible and designed to specifically target individuals who have varying levels of motivation to quit with treatment content specifically designed to meet their unique treatment needs over an extended treatment period. If found efficacious, MAPS + is likely to represent a sustainable, low-burden and engaging approach with broad reach and dissemination potential. For example, MAPS + could be disseminated within a variety of outreach programs and community-based networks targeting individuals with a history of CIN or cervical cancer. An important strength of our study is that individuals who are not yet ready to set a quit date are eligible to enroll. Two-thirds (i.e., 67%) of participants in our prior trial reported that they were not ready to quit at the time of study enrollment, [15] highlighting the importance of engaging these individuals in tobacco cessation treatment in an acceptable way. The Wellness Program component within MAPS + allows individuals who are not yet ready to commit to quitting smoking to focus on other life issues that are particularly salient for them (e.g., stress, family issues, finances, adjustment to a cancer diagnosis, fear of developing cancer or cancer recurrence).

In summary, theoretically based, sustainable, and tailored cessation treatments for cervical cancer and CIN survivors are needed. The MAPS + intervention approach that is under evaluation in the current trial was designed to build upon promising results from our recently completed MAPS trial among individuals with a history of CIN or cervical cancer. The current novel SMS-delivered text-based treatment adjuvant holds tremendous promise to deliver high-quality, theoretically based extended treatment designed to facilitate sustained long-term abstinence. Furthermore, given that cell phone ownership is nearly ubiquitous in the US, mobile phone-based treatments are likely to have tremendous reach and may represent an ideal treatment modality for vulnerable populations.

## Data Availability

The datasets generated and/or analyzed during the current study will be available from the corresponding author on reasonable request.

## Abbreviations

CIN: cervical intraepithelial neoplasia
GEE: generalized estimating equations
HPV: human papillomavirus
MAPS: Motivation And Problem Solving
MAPS+: Motivation And Problem Solving+
MNAR: missing not at random
NRT: nicotine replacement therapy
RCT: randomized clinical trial
REDCap: Research Electronic Data Capture
SES: socioeconomic status
SRNT: Society for Research on Nicotine and Tobacco
ST: Standard treatment
